# Treatment of patients with type 2 diabetes and non-alcoholic fatty liver disease: current approaches and future directions

**DOI:** 10.1007/s00125-016-3952-1

**Published:** 2016-04-21

**Authors:** Kenneth Cusi

**Affiliations:** Division of Endocrinology, Diabetes and Metabolism, University of Florida, 1600 SW Archer Road, Room H-2, Gainesville, FL 32610 USA; Malcom Randall Veterans Administration Medical Center, Gainesville, FL USA

**Keywords:** Fatty liver, Insulin resistance, NAFLD, NASH, Non-alcoholic steatohepatitis, Pioglitazone, Prediabetes, Treatment, Type 2 diabetes

## Abstract

Non-alcoholic fatty liver disease (NAFLD) is reaching epidemic proportions in patients with type 2 diabetes. Patients with NAFLD are at increased risk of more aggressive liver disease (non-alcoholic steatohepatitis [NASH]) and at a higher risk of death from cirrhosis, hepatocellular carcinoma and cardiovascular disease. Dysfunctional adipose tissue and insulin resistance play an important role in the pathogenesis of NASH, creating the conditions for hepatocyte lipotoxicity. Mitochondrial defects are at the core of the paradigm linking chronic excess substrate supply, insulin resistance and NASH. Recent work indicates that patients with NASH have more severe insulin resistance and lipotoxicity compared with matched obese controls with only isolated steatosis. This review focuses on available agents and future drugs under development for the treatment of NAFLD/NASH in type 2 diabetes. Reversal of lipotoxicity with pioglitazone is associated with significant histological improvement, which occurs within 6 months and persists with continued treatment (or for at least 3 years) in patients with prediabetes or type 2 diabetes, holding potential to modify the natural history of the disease. These results also suggest that pioglitazone may become the standard of care for this population. Benefit has also been reported in non-diabetic patients. Recent promising results with glucagon-like peptide 1 receptor agonists have opened another new treatment avenue for NASH. Many agents in Phase 2-3 of development are being tested, aiming to restore glucose/lipid metabolism, ameliorate adipose tissue and liver inflammation, or to inhibit liver fibrosis. By targeting a diversity of relevant pathways, combination therapy in NASH will likely provide greater success in the future. In summary, increased clinical awareness and improved screening strategies (as currently done for diabetic retinopathy and nephropathy) are needed, to translate recent treatment progress into early treatment and improved quality of life for patients with type 2 diabetes and NASH. This review summarises a presentation given at the symposium ‘The liver in focus’ at the 2015 annual meeting of the EASD. It is accompanied by two other reviews on topics from this symposium (by John Jones, DOI: 10.1007/s00125-016-3940-5, and by Hannele Yki-Järvinen, DOI: 10.1007/s00125-016-3944-1) and a commentary by the Session Chair, Michael Roden (DOI: 10.1007/s00125-016-3911-x).

## Why treat NASH in patients with type 2 diabetes?

Non-alcoholic fatty liver disease (NAFLD) is a frequent comorbidity in both paediatric and adult populations, in particular in the setting of obesity and type 2 diabetes [1–3]. It is estimated that between 75 million to 100 million individuals in the USA may have NAFLD [2], with high rates also reported worldwide [1]. The magnitude of the epidemic will make screening imperative, particularly in obese patients with type 2 diabetes, who are at the highest risk of developing its more aggressive form with hepatocyte injury (NASH). Patients with diabetes are also at a higher risk of fibrosis, end-stage liver disease and hepatocellular carcinoma (HCC), as well as extra-hepatic complications [4]. However, few studies have systematically screened patients with type 2 diabetes. In our experience, about 70% of obese patients with diabetes have NAFLD and as many as 30–40% have NASH [5–7]. The prevalence of both remains high even when plasma aminotransferase concentration is normal, with about half having steatosis (when measured by proton magnetic resonance imaging and spectroscopy [^1^H-MRS]), about one-third having NASH and many early fibrosis [8]. Other investigators have reported similarly high rates of steatosis (~70%) [9–12] and fibrosis (17–55%) [11–13]. The results of two recent large screening studies (one from Hong Kong [*n* = 1,918] [13] and another from Rotterdam [*n* = 3,041] [14]) were consistent with this, reporting that fibrosis affects one out of every six middle-aged patients with diabetes. Of note, on histology, isolated steatosis (i.e. without features of hepatocyte necrosis or inflammation) is no longer considered a ‘benign’ condition, at least in type 2 diabetes, as emerging evidence indicates that many patients with isolated steatosis develop hepatocyte injury and fibrosis over time [15]. Liver fibrosis is the single best predictor of future cirrhosis [16, 17] and it occurs much more frequently in diabetes [18].

It should also be noted that NAFLD is becoming a major cause of HCC in the USA. A recent study reported that between 2004 and 2009, HCC related to NASH increased by 9% annually and was associated with shorter survival time compared with other predisposing aetiologies [19]. Lack of systematic screening and treatment for NASH, even among hepatologists [20], has led to it being massively underdiagnosed, which explains why NASH is the second largest cause of cirrhosis and liver transplantation in the USA [21].

Another major reason for addressing NAFLD in diabetes is its strong association with cardiovascular disease (CVD) [1, 2, 22]. While most physicians place a high priority on preventing macrovascular complications in type 2 diabetes, few are aware that the presence of NAFLD appears to significantly increase the risk. Although the nature of this association remains a subject of intense investigation, there is good evidence that NAFLD promotes dyslipidaemia [23], hyperinsulinaemia [24] and subclinical inflammation [1, 2, 22], all of which are potentially atherogenic risk factors. The presence of NAFLD may also worsen microvascular disease and other comorbidities often present in diabetes [1, 4].

In summary, patients with type 2 diabetes who also have NASH appear to be at a significantly higher risk of death from either cirrhosis, HCC and/or CVD. A specific screening strategy for this population must be developed and implemented, as has been done for diabetic microvascular complications [1, 25], and included in future guidelines [26, 27].

## Available pharmacological agents for the treatment of NASH in patients with type 2 diabetes

### Insulin-sensitisers in NASH

Restoring insulin action is a major treatment target given the central role of adipose tissue insulin resistance and ‘lipotoxicity’ in the pathogenesis of NASH [1]. Insulin sensitivity and hepatic steatosis usually improve with lifestyle intervention in proportion to the magnitude of weight loss. Significant reversal of hepatocyte injury requires weight loss of about 10% [1, 2, 28]. Bariatric surgery also improves NASH [28]. In contrast, long-term improvement of fibrosis by either lifestyle intervention or bariatric surgery is uncertain owing to a lack of controlled prospective studies beyond 12 months’ duration (reviewed in [26, 28]).

Insulin-sensitisers have been widely tested in NAFLD. Early open-label studies with metformin suggested a histological benefit [29, 30]. However, a specific effect of the biguanide was difficult to establish given the larger than expected associated weight loss in these studies, while more recent randomised controlled trials (RCTs) have yielded negative results in both children [31] and adults (reviewed in [1, 3, 26, 28]). While the aetiology of NASH is multifactorial, unabated lipolysis in dysfunctional/insulin-resistant adipose plays a key role by promoting ectopic triacylglycerol accumulation in many tissues [1–3]. Adipose tissue dysfunction and hepatic insulin resistance are more severe in diabetic patients with NASH compared with matched obese controls with only isolated steatosis [32], and mitochondrial defects appear to be at the core of the paradigm linking excess substrate supply, insulin resistance, lipotoxicity and NASH [33]. Recent studies from our laboratory [34, 35], and others [36–38], support this view. In animal models of diet-induced NASH (C57BL/6 mice fed a high-*trans*-fat, high-fructose diet for 24 weeks) hepatic mitochondrial fluxes are increased, but not enough to prevent the formation of inflammatory cytokines, lipid peroxidation products and other toxic lipid metabolites (ceramides, diacylglycerols and acylcarnitines, among others), suggesting inadequate adaptation with inefficient disposal of excess fatty acids through the tricarboxylic acid (TCA) cycle [34]. Recent human studies indicate inadequate mitochondrial adaptation despite increased TCA cycle activity in obese patients with hepatic steatosis [39]. In patients with NASH, mitochondrial mass is greater but is characterised by proton leakage, reduced antioxidant capacity and more prone to trigger inflammation [38]. In these provocative studies only a handful of patients were studied, so much more remains to be learned in relation to factors such as ethnicity, presence or absence of diabetes, ageing, genetic polymorphisms (e.g. *PNLPA3*, *TM6SF2*) and response to weight loss or pharmacotherapy. It has been established that mitochondrial oxidative capacity must retain its ability to be highly adaptable (retain ‘metabolic flexibility’) to changing metabolic demands in order to prevent cell injury and activation of apoptotic pathways. Under conditions of chronic overfeeding, as observed in obesity or type 2 diabetes, metabolic flexibility is lost. This hypothesis clearly requires further experimental validation. However, recent proof-of-concept experimental approaches that relieve mitochondrial demand either by weight loss or hormonal manipulation [40], or more specifically, by mild mitochondrial uncoupling using the protonophore 2,4-dinitrophenol (DNP) [36], reverse hepatic triacylglycerol accumulation and NASH.

Within this context, thiazolidinediones (TZDs) are ligands that target the transcription factor peroxisome proliferator-activated receptor (PPAR)-γ, with broad effects on glucose/lipid metabolism through modulation of substrate supply, insulin signalling and mitochondrial fatty acid oxidation [1, 41]. In humans with NASH, pioglitazone enhances insulin sensitivity and prevents excessive rates of lipolysis [42–44]. The positive effects of PPAR-γ action on adipocyte biology translate into a two- to threefold increase in plasma adiponectin concentration [44]. In recent work, Bril et al [45] reported that the response to pioglitazone in NASH can be predicted by the increase in plasma adiponectin levels within the first 1–3 months after treatment initiation. It has been about a decade since Belfort et al [42] first reported in a proof-of-concept study that in patients with NASH and either impaired glucose tolerance or type 2 diabetes, pioglitazone treatment significantly ameliorated hepatic steatosis and necroinflammation. The NAS improved for most participants, while treatment difference in resolution of NASH vs placebo occured in about one-third. Pioglitazone also reduced liver fibrosis compared with baseline (*p* = 0.002), but not when compared with placebo (*p* = 0.08). This RCT was important in establishing that NASH could be reversed within a relatively short period of time (6 months). Pioglitazone also prevents the progression from impaired glucose tolerance to diabetes [46], and one may speculate that this effect is tied to its ability to reverse NAFLD/NASH [47]. Histological improvement with pioglitazone in NASH was later also reported in patients without diabetes [48, 49]. Recently, a 3 year study in 101 patients with NASH and either impaired fasting glucose and/or impaired glucose tolerance or type 2 diabetes confirmed its long-term safety and efficacy in this population (K. Cusi, B. Orsak, F. Bril, R. Lomonaco, J. Hecht, C. Ortiz-Lopez, unpublished results). Taken together, these results suggest that pioglitazone may modify the natural history of the disease. No patient discontinued the trial due to a pioglitazone-induced adverse event and weight gain was modest overall (3 kg over 3 years). Weight gain with pioglitazone treatment is primarily due to improved insulin action and enhanced adipocyte triacylglycerol storage, and less often the result of fluid retention [50, 51]. Pioglitazone decreases cardiovascular events [1, 41], a feature frequently forgotten but of value in this population. However, development of shortness of breath or congestive heart failure, while rare, may occur in patients with type 2 diabetes and NAFLD with undiagnosed diastolic dysfunction [52]. Bone loss may occur with TZD treatment in women, and pioglitazone should not be used in pregnancy or in children or adolescents [53]. A 10 year prospective study showed no association between pioglitazone and bladder cancer [54].

### Glucagon-like peptide 1 receptor agonists and dipeptidyl peptidase 4 inhibitors

Indirect evidence from animal studies (reviewed in [1, 55]), and recent meta-analysis of clinical trials of liraglutide in type 2 diabetes [56], have suggested that glucagon-like peptide 1 receptor agonists (GLP-1RAs) could improve NASH. However, clear evidence was lacking until the landmark proof-of-concept study by Armstrong et al [57]. The investigators treated 52 obese insulin-resistant patients, one-third of whom had type 2 diabetes, with liraglutide or placebo for 48 weeks. Resolution of NASH was observed in 39% of liraglutide-treated patients compared with 9% of patients in the placebo group (RR 4.3, 95% CI 1.0, 17.7; *p* = 0°019), a treatment difference of ~30%. In addition, there was less progression of liver fibrosis over time in the treatment group (*p* < 0.05). Liver and adipose tissue insulin sensitivity improved in a subset of patients that underwent careful glucose turnover studies [58]. These results have created significant excitement for additional GLP-1RA studies in NASH to understand the relative contribution of unspecific mechanisms such as changes in weight, insulin sensitivity or glucotoxicity [59] vs effects on specific hepatocyte GLP-1 signalling pathways [60, 61].

In contrast, studies with dipeptidyl peptidase 4 (DPP-4) inhibitors have reported mixed results. Sitagliptin has been reported to be neutral [62] or to decrease [63] plasma aminotransferases, although their overall impact on liver histology is unknown. In diabetic patients with mild steatosis, Macauley et al [64] observed a small but significant reduction (from 7.3% to 5.3%, normal ≤5.5%; *p* = 0.001) in hepatic triacylglycerol accumulation with vildagliptin treatment for 6 months, a change that correlated closely with the decrease in fasting plasma concentration. Thus, hyperglycaemia may be an important factor that promotes hepatic steatosis, as suggested in cross-sectional studies [8, 32], although the role of reversing glucotoxicity per se on NASH (independent of treating insulin resistance) has never been examined in an RCT in type 2 diabetes.

### Sodium–glucose cotransporter 2 (SGLT2) inhibitors

Control of hyperglycaemia by inhibition of proximal tubule glucose reabsorption [65] is actively being investigated as a potential approach for NAFLD in patients with type 2 diabetes. A reduction in intrahepatic triacylglycerol accumulation would be expected from a decrease in substrate supply to the liver by the combined effects of normoglycaemia plus modest weight loss and enhanced insulin sensitivity. In animal models of NASH, sodium–glucose cotransporter 2 (SGLT2) inhibitors have unique antifibrotic properties [66]. In patients with diabetes, levels of plasma aminotransferases decrease during treatment with SGLT2 [67, 68]. Recently, pooled data from four 26 week placebo-controlled studies of canagliflozin (*n* = 2313) and two 52 week active-controlled studies of canagliflozin vs sitagliptin (*n* = 1488) found significant reductions in plasma alanine aminotransferase (ALT) with canagliflozin 300 mg compared with placebo or sitagliptin [69]. Changes in aspartate aminotransferase (AST)/ALT were fully explained by the reduction in HbA_1c_ and body weight. Future studies are needed to define the role of SGLT2 inhibitors in the management of patients with NAFLD and type 2 diabetes.

### Other therapeutic agents tested for the treatment of NASH

Many agents have been tested for the treatment of NASH [1–3, 28, 55] but few have included a significant number of patients with type 2 diabetes. Most have not improved liver histology. Lipid-lowering agents have been tested extensively and can be used safely in this population [26, 27], but statins [70], ezetimibe [71], fibrates [72], niacin [72], omega-3 polyunsaturated fatty acids (PUFAs) [73] and colesevelam [74] do not improve steatosis to a clinically meaningful extent (or hepatocyte injury when examined; reviewed in [28, 55]). Instead, amelioration of hepatocyte injury has been reported with vitamin E in adults without type 2 diabetes at doses of 800 IU/day [49]. However, resolution of NASH was of borderline significance (36% vs 21%, *p* = 0.05 vs placebo) in contrast to the effect of pioglitazone in the same trial (47% vs 21%, *p* = 0.001 vs placebo) [49]. In a controlled trial in non-diabetic children with NASH, treatment with vitamin E or metformin had overall negative effects [31]. Vitamin E may decrease intracellular oxidative stress, although the mechanism of action is unclear. The long-term safety of vitamin E in patients with NASH remains to be established given its controversial increase in the risk of prostate cancer with long-term exposure [75]. An ongoing RCT will soon establish its role in patients with type 2 diabetes, either alone or combined with pioglitazone (NCT01002547). Finally, pentoxifylline, a TNF-α agonist and non-selective phosphodiesterase inhibitor, has been studied in NASH in two small controlled 12 month studies. Van Wagner et al [76] reported no histological improvement in 30 patients, while another small trial (*n* = 26) observed a modest benefit on steatosis, lobular inflammation and fibrosis [77]. Larger studies are clearly needed to establish its role in NASH.

## Future treatments for patients with NASH

From the previous sections it is evident that additional agents are needed for the treatment of NASH. Moreover, no existing agent tested has been specifically approved to this end. This has led to an explosion of agents currently under investigation. Importantly, most studies now include a large number of patients with type 2 diabetes given their higher risk of disease progression.

Many approaches have been tested. Table 1 includes agents at more advanced stages of development (Phase 2a/2b or early Phase 3 trials). But the challenge ahead is substantial. For example, attempts to control triacylglycerol synthesis, such as with 11β-hydroxysteroid dehydrogenase type 1 (11β-HSD1) [78] or stearoyl coenzyme A desaturase (SCD)-1 pathways [79] have led to overall discouraging results. Greater success has been achieved by targeting PPARα/PPARδ with the dual agonist elafibranor (GFT505) [80], a partial PPARα agonist with about 60% of the maximal response (E_max_) of fenofibrate [81]. Dual PPARα and PPARδ activation mitigates inflammation and improves hepatic insulin sensitivity in vivo [82] and in insulin-resistant patients [83]. In contrast, the PPARα agonist fenofibrate has not been associated with a decrease in insulin resistance or plasma aminotransferase concentration in patients with the metabolic syndrome [84] or decreased hepatic steatosis in patients with NAFLD [72]. Despite the above strong rationale, discouraging results were reported after 52 weeks of elafibranor treatment in 276 patients with biopsy-proven NASH (~40% with type 2 diabetes) as it failed to meet the primary outcome of resolution of NASH without worsening of fibrosis [84]. This was, at least in part, due to many patients having borderline NASH on liver histology before treatment. However, resolution of NASH (20% vs 11%, *p* = 0.018) and improved scores of hepatocyte injury and fibrosis were noted at the higher dose (120 mg/day) in patients with more severe disease at baseline (NAFLD activity score [NAS] ≥4; *n* = 234) [85]. These results have been the basis for an ongoing large multicentre RCT with elafibranor enrolling only patients with more advanced liver disease (NAS ≥ 4) (NCT02704403) [85].

**Table 1:** Pharmaceutical agents under development for the treatment of NASH

Therapeutic agent	Manufacturer	Target	Proposed mode of action
BMS986036	Bristol-Myers Squibb	Modulation of FGF21 metabolism	Improvement of hepatic lipid and glucose metabolism; anti-inflammatory
Cenicriviroc	Tobira Therapeutics	CCR2 and CCR5	Inhibition of CCR2- and CCR5-mediated monocyte/macrophage infiltration and inflammation
Elafibranor	Genfit	Modulation of hepatic PPARα and PPARδ pathways	Stimulation of NEFA oxidation; improvement of lipid and glucose metabolism; prevention of inflammation
Emricasan	Conatus Pharmaceuticals	Caspase pathways (pan-caspase inhibitor)	Inhibition of fibrosis by blocking caspase protease activation and apoptosis pathways
GR-MD-02	Galectin Therapeutics	Galectin-3 inhibitor	Prevention of inflammation and fibrosis
Obeticholic acid	Intercept Pharmaceuticals	FXR agonist	Regulation of hepatic glucose and lipid metabolism
Px-104	Phenex Pharmaceuticals/ Gilead Sciences	FXR agonist	Regulation of hepatic glucose and lipid metabolism
Simtuzumab	Gilead Sciences	LOXL2 enzyme activity	Inhibition of fibrosis by a LOXL2 monoclonal antibody

Agents are listed in alphabetical order

FXR, Farnesoid X receptor; LOXL2, Lysyl oxidase-like 2

A different strategy involves more directly targeting inflammation by inhibition of C-C chemokine receptors type 2 (CCR2) and type 5 (CCR5), which mediate monocyte and macrophage infiltration and inflammation in adipose tissue [86, 87]. Overexpression of CCR2 and CCR5 receptors and their ligands in adipose tissue of obese patients is associated with increased inflammation and insulin resistance. Cenicriviroc is a potent inhibitor of ligand binding of CCR2 and CCR5 with antifibrotic properties in animal models of liver/kidney fibrosis. Post hoc analysis of Phase 2b studies in HIV-1 patients revealed that cenicriviroc treatment improved plasma biomarkers of fibrosis (AST to Platelet Ratio Index [APRI] and Fibrosis 4 calculator [FIB-4] scores). Its mechanism of action in humans is being actively studied in obese, insulin-resistant individuals at risk of NAFLD (ORION study; NCT02330549), and in a large controlled multicentre trial in patients with NASH who are at increased risk of disease progression due to the presence of ≥1 risk factor such as the metabolic syndrome, type 2 diabetes or hepatic fibrosis (CENTAUR; NCT02217475) [88].

Given that fibrosis is the primary determinant of long-term adverse outcomes [16, 17], several novel agents are specifically targeting fibrosis-related pathways. Approaches include inhibition of apoptotic pathways with the pan-caspase inhibitor emricasan, a compound that has been recently granted fast track designation by the United States Food and Drug Administration and is being tested in a Phase 2b clinical trial in NASH cirrhosis (EmricasaN, a Caspase inhibitOR, for Evaluation [ENCORE-NF]). Another strategy is treatment of advanced fibrosis targeting galectins, a family of proteins with high affinity to galactose-containing glycoproteins present on cell surfaces and the extracellular matrix [89]. Galectin-3 protein is highly expressed in immune cells and has been associated with inflammation and fibrosis in several disease models, including hepatic fibrosis. A Phase 2a multicentre trial is underway in individuals with portal hypertension and NASH cirrhosis (NCT02462967). Other approaches in Phase 2 studies aimed at controlling fibrogenesis include targeting growth and inflammation through the fibroblast growth factor 21 (FGF21) pathway [90] (compound BMS986036, NCT02413372), or preventing collagen cross-linking by inhibiting lysyl oxidase-like 2 (LOXL2) enzyme activity with the monoclonal antibody simtuzumab (being tested in NASH patients with [NCT01672879] or without [NCT01672866] cirrhosis).

Finally, histological improvement may be achieved in NASH by modulation of hepatic glucose and lipid metabolism through farnesoid X receptor (FXR) pathways [91], as recently shown with the bile acid derivative 6-ethylchenodeoxycholic acid (obeticholic acid) [92]. The primary outcome of a decrease in the NAS by ≥2 without worsening of fibrosis was reached in 45% of patients treated with obeticholic acid vs 21% on placebo (RR 1.9, 95% CI 1.3, 2.8; *p* = 0.0002; treatment effect ~24%). However, to reach this prespecified primary endpoint, change in the NAS did not have to necessarily include ballooning or two separate NAS variables. This departure from prior trials by the NASH Clinical Research Network [31, 49], in part aimed at simplifying the primary outcome, has made comparisons with other RCTs difficult [31, 49, 84]. While resolution of NASH did not change significantly (22% vs 13% on placebo, *p* = 0.08), individual histological outcomes improved compared with placebo: steatosis 61% vs 38% (*p* = 0.001), inflammation 53% vs 35% (*p* = 0.006), ballooning 46% vs 31% (*p* = 0.03) and fibrosis 35% vs 19% (*p* = 0.004), giving a treatment difference vs placebo of between ~15% and 23%. There has been some concern about the increase from baseline in plasma LDL-cholesterol and the reduction in HDL-cholesterol levels, as well as the observed worsening of insulin sensitivity (according to HOMA-IR). Of note, changes in fasting plasma insulin during the trial were confounded, at least in part, by patients with diabetes on exogenous insulin during the study. However, a modest improvement in hepatic insulin sensitivity is observed with short-term use of obeticholic acid [93] at the daily dose of 25 mg used in the Farnesoid X Receptor Ligand Obeticholic Acid in NASH Treatment (FLINT) [92]. A larger multicentre study is underway to fully assess its long-term efficacy and safety.

## Conclusions: perspective for the future

Today there is a much greater awareness of the severe metabolic and liver-specific complications associated with NASH. Within this context, there is growing consensus to replace the acronym ‘NASH’, a disease of ‘not being alcoholic steatohepatitis’ with a more descriptive name that would help clinicians conceptually grasp the condition to be diagnosed and treated [1, 94, 95]. As reviewed earlier, there are many intracellular pathways that contribute to hepatocyte injury, and the heterogeneous nature of the disease makes a unifying name difficult. However, this problem is also true for most chronic diseases, including type 2 diabetes. A name that highlights the central role of hepatocyte lipotoxicity secondary to adipose tissue insulin resistance characteristic of obesity would be fitting. Lipotoxicity primes cells to mitochondrial dysfunction and makes them more vulnerable to toxic lipid metabolites, oxidative stress and activation of multiple inflammatory pathways [1, 94]. Future work needs to deepen our understanding of the crosstalk between liver and adipose tissue, but the name ‘lipotoxic liver disease’ would encompass a significant aspect of the disease and serve as a reminder to clinicians about the important role of dysregulated adipose tissue in NAFLD.

Specific screening and treatment recommendations for patients with type 2 diabetes and NASH will need to be developed and actively pursued in the clinic. Screening for NASH in type 2 diabetes, particularly in obese patients, should be encouraged as is currently done for microvascular complications of diabetes such as retinopathy, neuropathy and nephropathy. Only early intervention is likely to modify the natural history of the disease and halt the growing epidemic of NASH [21]. Recent studies support such an approach, as one in six middle-aged patients with type 2 diabetes have liver fibrosis [13, 14, 25]. There is recent evidence of a long-term safe and effective treatment for this population in pioglitazone (K. Cusi et al, unpublished results), which will become to NASH what metformin is to type 2 diabetes today—first-line therapy and the background agent for combination strategies. This should not undermine the search for other pharmacological agents targeting the many dysfunctional pathways in NASH discussed above. Of note, drug development should target resolution of NASH, not isolated steatosis, although in early exploratory studies significant reduction in liver triacylglycerol content on imaging (^1^H-MRS) may potentially predict improvement in NASH. The magnitude of improvement on ^1^H-MRS imaging that translates into resolution of NASH is unknown at the present time, but is likely to depend on the mechanism of action of a specific compound. As new agents become available, it is likely that combination therapy will become widely accepted for such a heterogeneous disease such as NASH, as is common practice for the treatment of type 2 diabetes or hypertension.

Future studies should explore the role of glycaemic control per se in NASH, as has been investigated for microvascular complications of diabetes. No trial to date has addressed this issue. There is also a need for long-term RCTs on the optimal lifestyle intervention, how to improve adherence and on the best macronutrient composition for NASH in type 2 diabetes. The additional role of weight loss agents and of bariatric surgery need to be better defined prospectively in controlled studies. Drug development trials will need more consistency in their trial design and primary outcomes, as well as improved research networks for efficient recruitment into large multicentre RCTs. Better standardisation across imaging methodologies and biomarkers, with better correlation/validation with histology, will be also imperative for the screening of large cohorts and the assessment of treatment response.

In summary, while the diagnosis of NAFLD/NASH today remains a challenge, screening with a combination of validated novel genetic, imaging and plasma biomarkers will in the near future facilitate management. We are at an exciting time where new awareness about the impact of NASH will prompt earlier diagnosis and treatments that for the first time may alter the natural history of the disease and improve the quality of life of millions of patients.

## Abbreviations

AST: Aspartate aminotransferase
CCR: C-C chemokine receptor
CVD: Cardiovascular disease
GLP-1: Glucagon-like peptide 1
GLP-1RAs: GLP-1 receptor agonists
HCC: Hepatocellular carcinoma
NAFLD: Non-alcoholic fatty liver disease
NAS: NAFLD activity score
NASH: Non-alcoholic steatohepatitis
PPAR: Peroxisome proliferator-activated receptor
RCT: Randomised controlled trial
SGLT2: Sodium–glucose cotransporter 2
TCA: Tricarboxylic acid
TZD: Thiazolidinediones
